# Stepwise mini-incision microdissection testicular sperm extraction in NOA patients with a history of cryptorchidism: a case–control study

**DOI:** 10.1186/s12610-023-00196-w

**Published:** 2023-08-17

**Authors:** Shuai Xu, Yuhua Huang, Chencheng Yao, Peng Li, Erlei Zhi, Wei Chen, Cunzhong Deng, Fujun Zhao, Zheng Li, Ruhui Tian

**Affiliations:** Department of Andrology, Center for Men’s Health, Department of ART, Institute of Urology, Urologic Medical Center, Shanghai Key Laboratory of Reproductive Medicine, Shanghai General Hospital, Shanghai Jiao Tong University School of Medicine, Shanghai, China

**Keywords:** Stepwise mini-incision microdissection testicular sperm extraction, Microdissection testicular sperm extraction, Non-obstructive azoospermia, Cryptorchidism, Sperm retrieval rate, Extraction de Spermatozoïdes testiculaires par Microdissection avec Mini-incision par étapes, Extraction de Spermatozoïdes testiculaires par Microdissection, Azoospermie non obstructive, Cryptorchidie, Taux de Récupération de Spermatozoïdes

## Abstract

**Background:**

Although the orchiopexy is recommended for cryptorchidism to preserve male fertility, non-obstructive azoospermia (NOA) may occur in adulthood. Fortunately, a great many of azoospermic men may obtain sperm by microdissection testicular sperm extraction (mTESE). Due to the potential injuries caused by testicular diagnostic biopsy and vascular damage at the time of orchidopexy, minimal invasiveness is particularly important during mTESE, aims to reduce the surgical damage and avoids secondary testicular failure. This comparative study aims to investigate the efficacy of stepwise mini-incision mTESE technique by comparison with standard mTESE in the treatment of NOA patients with a history of cryptorchidism.

**Results:**

A total of 73 mTESE procedures were divided into two groups: Group 1 included 37 cases performed by stepwise mini-incision mTESE, while Group 2 included 36 cases with standard mTESE. The overall sperm retrieval rate (SRR) in the two groups was 68.5% (50/73), with no significant difference in SRR between Group 1 (78.4%, 29/37) and Group 2 (58.3%, 21/36) (*P* = 0.1). In addition, 46.0% of the patients (17/37) obtained sperm in the first mini-incision step in Group 1, which was also equal to an overall SRR in Group 2 (58.3%, 21/36) (*P* = 0.3). The operation time in Group 1 (72.6 ± 33.9 min) was significantly shorter than that in Group 2 (90.4 ± 36.4 min) (*P* = 0.04). Patients with an orchidopexy age no more than 10 years old had a higher SRR (79.5%, 31/39) than others (55.9%, 19/34) (*P* = 0.03). There were no postoperative complications including wound infection, scrotal hematoma, persistent pain, and testicular atrophy during a follow-up period of at least 6 months.

**Conclusions:**

In conclusion, our study suggests that the stepwise mini-incision mTESE could be a promising approach for sperm retrieval in NOA men with a history of cryptorchidism. While the technique may potentially reduce operation time and surgical invasiveness, further research is needed to validate these findings on a larger scale. The results also suggest that age at orchidopexy may affect SRR and have important implications for the management of cryptorchidism.

## Résumé

**Contexte:**

Bien que l’orchidopexie soit recommandée en cas de cryptorchidie afin de préserver la fertilité masculine, une azoospermie non obstructive (NOA) peut survenir à l’âge adulte. Heureusement, un grand nombre d’hommes azoospermiques peuvent obtenir des spermatozoïdes lors d’une extraction de spermatozoïdes testiculaire par microdissection (mTESE). En raison des potentielles lésions causées par la biopsie diagnostique testiculaire et des lésions vasculaires survenant au moment de l’orchidopexie, une approche minimalement  invasive est particulièrement importante pendant la mTESE;  elle vise à réduire les dommages chirurgicaux et à éviter une insuffisance testiculaire secondaire. La présente étude comparative a pour but d’ étudier l’efficacité de la mTESE par mini-incision par étapes en comparaison avec la mTESE standard dans le traitement des patients NOA qui ont des antécédents de cryptorchidie.

**Résultats:**

Au total, 73 procédures de mTESE ont été divisées en deux groupes: le Groupe 1 comprenait 37 cas effectués avec la mTESE par mini-incision par étapes, tandis que le Groupe 2 comprenait 36 cas réalisés par la mTESE standard. Le taux global de récupération de spermatozoïdes (SRR) dans les deux groupes était de 68, 5% (50/73), sans différence significative de SRR entre le Groupe 1 (78, 4%, 29/37) et le Groupe 2 (58, 3%, 21/36) (*P* = 0,1). De plus, 46% des patients (17/37) ont obtenu des spermatozoïdes lors de la première étape de mini-incision dans le Groupe 1, ce qui était identique au SRR global dans le Groupe 2 (58%, 21/36) (*P* = 0,3). Le temps opératoire du Groupe 1 (72, 6 ± 34 min) était significativement plus court que celui du Groupe 2 (90, 4 ± 36 min) (*P* = 0,04). Les patients dont l’orchidopexie avait été réalisée au plus tard à l’âge de 10 ans avaient un SRR plus élevé (79, 5%, 31/39) que les autres (55, 9%, 19/34) (*P* = 0,03). Il n’y a pas eu de complications postopératoires, que ce soit infection de la plaie, hématome scrotal, douleur persistante, ou atrophie testiculaire pendant une période de suivi d’au moins 6 mois.

**Conclusions:**

Notre étude suggère que la mTESE par mini-incision par étapes pourrait être une approche prometteuse pour la récupération de spermatozoïdes chez les hommes NOA ayant des antécédents de cryptorchidie. Bien que la technique puisse potentiellement réduire le temps d’opération et le caractère invasif du geste chirurgical, des recherches supplémentaires sont nécessaires pour valider ces résultats à plus grande échelle. Les résultats suggèrent également que l’âge à l’orchidopexie peut affecter le SRR et avoir des implications importantes pour la prise en charge de la cryptorchidie.

## Background

Non-obstructive azoospermia (NOA), defined as the absence of sperm in the ejaculate secondary to impaired spermatogenesis, is the most severe form of male factor infertility accounting for about 10–15% of infertile men [1]. NOA may be caused by a variety of etiologies, including genetic defects, cryptorchidism, post-pubertal mumps orchitis, gonadotoxic effects from medications/radiation, and other unknown causes currently classified as idiopathic [2].

Testicular sperm retrieval combined with intracytoplasmic sperm injection (ICSI) has been the first-line treatment on NOA. Microdissection testicular sperm extraction (mTESE) is widely recommended for sperm retrieval, as this method enables the dilated tubules more likely to contain foci of intact spermatogenesis to be identified under microscopic visualization [3]. Due to the larger incision and the facility of operating microscope, mTESE demonstrates an absolute higher sperm retrieval rate (SRR) than conventional or multifocal testicular sperm extraction (TESE) [4]. While the mTESE technique involves a meticulous microsurgical exploration of the testicular parenchyma, the invasiveness of surgical procedures has increased. Therefore, the safety concerns, such as surgically induced devascularization and hypogonadism, have been given more and more attention [5, 6]. Some scholars advocated a stepwise approach during testicular sperm retrieval, in which a mini-incision mTESE is initially performed, followed by a standard mTESE using the enlarged testicular incision if the previous step fails [7–9]. The stepwise approach makes sense, because a significant subset of the men had sperm identified in the superficial tissue and/or only required a unilateral mTESE [10]. In addition, it particularly applicable for cryptorchidism patients, whose testes may have already suffered damages during the orchiopexy.

Cryptorchidism, one of the most common congenital anomalies, is a pathological condition in which the testis fails to descend to the scrotum [11]. Numerous studies have shown that the history of cryptorchidism was associated with a high risk of NOA or lack of germ cells in adult men [12]. Orchiopexy is recommended for testes that remain undescended after six months of age [13]. Despite the best efforts, the incidence of NOA in patients was 25 times more often than the control population [14]. Fortunately, a great many of azoospermic men who underwent orchiopexy may obtain sperm by the technique of testicular sperm retrieval [15].

Here we conducted a retrospective study to identify the efficacy of stepwise mini-incision mTESE technique in the treatment of NOA patients with a history of cryptorchidism by comparison with standard mTESE.

## Methods

### Patients

We performed a retrospective analysis, which aims to investigate the efficacy of stepwise mini-incision mTESE technique by comparison with standard mTESE in the treatment of NOA patients with a history of cryptorchidism. A total of 73 mTESE procedures were performed in NOA patients with a history of cryptorchidism at Shanghai General Hospital during March 2015 and August 2021. We routinely informed the patients of different options before surgery, and the patients decided which method to use. According to different surgical methods, we divided all cases into two groups. Group 1 includes 37 cases performed by stepwise mini-incision mTESE, while Group 2 is standard mTESE, including 36 cases. All patients were diagnosed according to medical history, physical examination and supplementary examination. Clinical characteristics of all cases were collected, including ages, history of orchidopexy, serum levels of hormone, SRR and postoperative complications. The hormone levels were measured daily at 8:00 am. Normal ranges for adults were 1.3–19.3 IU/L (FSH), 1.2–8.6 IU/L (LH), 6.1–27.1 nmol/L(T). The semen analysis met the diagnostic criteria of WHO laboratory manual for the examination and processing of human semen (5th Edition) (i.e., no sperm are observed in the pellet obtained by centrifugation of the semen at 3000 g for 15 min at least twice) [16]. All patients were excluded from obstructive azoospermia, such as a history of epididymitis, absence of vas deferens, and ejaculatory duct obstruction, etc. Before proceeding to mini-incision TESE-ICSI or standard mTESA, each case was first reviewed by the clinical team (urologist, gynecologist, and embryologist). Couples with cryptozoospermia were offered three treatment options: (i) ICSI with fresh or previously frozen and thawed ejaculated spermatozoa, (ii) ICSI with fresh ejaculated spermatozoa and mTESA as a back-up if no viable ejaculated spermatozoa are found or (iii) mini-incision TESE- ICSI. All the couples were told of the potential risks of micro-TESE (bleeding, infection, pain, hypogonadism, irreversible testicular dysfunction).

### Histological examination

Histopathological evaluation of the testicular parenchyma was performed in all patients, and the degree of spermatogenesis was classified according to Johnsen score. Fresh testicular tissues from donors were fixed in 4% paraformaldehyde for 12–24 h at 4 °C, embedded in paraffin, and sectioned. Before staining, tissue sections were dewaxed in xylene, rehydrated using a gradient series of ethanol solutions, and washed in distilled water. Then the sections were stained with PAS/hematoxylin and dehydrated using increasing concentrations of ethanol and xylene. Sections were allowed to dry before applying neutral resin to the coverslips. The staining images were captured with a Nikon Eclipse Ti-S fluorescence microscope (Nikon). Johnsen score of testis sections was identified according to the previous study [17].

### mTESE

All surgical procedures were carried out under general anesthesia. Generally, the testis with larger volume, or the right side, or the testis born in a normal scrotal position was explored first. A longitudinal scrotal incision was made over the right or left scrotum (a contralateral incision was made if a bilateral procedure proved necessary), or the median raphe incision if available. ​The subcutaneous tissue was then gently dissected to expose the testis. The testis was delivered, but sometimes it was difficult due to scarring. For stepwise mini-incision mTESE, one to three mini-incision(s) were successively made in the equatorial region through the tunica albuginea to expose a small portion of testicular parenchyma under the operating microscope. The number of mini-incisions depended on the scarring and the exposure of tunica albuginea. The available testicular tissue beneath the mini-incision was examined under the operating microscope at 15 × to 24 × magnification to locate and collect dilated seminiferous tubules. The dilated tubules were collected and immediately evaluated by an embryologist available in the operating room. If no sperm were found beneath the mini-incision(s), the incision(s) was then extended to perform the standard mTESE. If no sperm was found in the initial side, the same procedure was carried out in the contralateral testis. The procedure was terminated when sperm were retrieved or when further dissection was considered likely to jeopardize the testicular blood supply. At the completion of the procedure, the tunica albuginea and skin were closed with 5–0 suture. In this study, the same surgeon performed all procedures.

For standard mTESE, the microdissection was started after a wide middle albugineal incision was made in the equatorial region. If no sperm was found in the initial side, the same procedure was carried out in the contralateral testis. Other procedures were similar to the stepwise mini-incision mTESE.

### Ethical approval

The study was approved by the Ethics Committee of Shanghai General Hospital (Number: 2020SQ041).

### Statistical analysis

IBM Statistical Package for the Social Sciences (SPSS, SPSS Inc, IBM Corp.) was used to collect data and perform statistical analysis. Mean ± standard deviation (Mean ± SD) was used for normal distribution data, and the median (*M* (P_25_, P_75_)) was used for nonnormal distribution data. The percentage was used to express the count data. The Student’s t-test was used to compare the mean of two independent samples that followed a normal distribution, whereas the nonparametric test was used to compare two independent samples that did not follow a normal distribution. The Pearson χ^2^ test was used to compare groups. All statistical tests were two sided, and *P* < 0.05 was considered statistically significance. The receiver operating characteristic (ROC) were plotted using the Medcalc software, and their area under the curve (AUC) were calculated.

## Results

### Clinical characteristics

The clinical characteristics of the patients are shown in Table 1. The patients’ characteristics of two groups were comparable at baseline.

**Table 1:** Clinical characteristics and hormonal values of the patients

Parameter	Total	Group 1	Group 2	*P*—value
**Number of men**	73	37	36	/
**Age (Mean ± SD, years)**	31.1 ± 3.8	31.2 ± 3.7	31.0 ± 3.9	0.8^a^
**Age at orchidopexy [*M*** **(P**_**25**_**, P**_**75**_**), years]**	10 (5, 23)	10 (6, 19)	9 (5, 23.3)	1.0^b^
**Unilateral cryptorchidism**	27.4% (20/73)	27.0% (10/37)	27.8% (10/36)	1.0^c^
**Bilateral cryptorchidism**	72.6% (53/73)	73.0% (27/37)	72.2% (26/36)	1.0^c^
**Semen Volume (Mean ± SD, ml)**	2.8 ± 0.5	2.7 ± 0.5	2.9 ± 0.5	0.3^a^
**FSH (Mean ± SD, IU/L)**	29.4 ± 17.9	27.2 ± 16.0	31.4 ± 19.3	0.4^a^
**LH (Mean ± SD, IU/L)**	16.0 ± 9.4	14.5 ± 8.6	17.4 ± 9.8	0.2^a^
**T (Mean ± SD, nmol/L)**	15.3 ± 7.9	15.7 ± 8.5	14.9 ± 7.4	0.7^a^
**Left testicular volume (Mean ± SD, ml)**	7.5 ± 2.9	7.9 ± 2.6	7.0 ± 3.2	0.2^a^
**Right testicular volume (Mean ± SD, ml)**	6.9 ± 2.9	7.3 ± 2.9	6.4 ± 2.8	0.2^a^

Patients were divided into two groups based on the type of surgery: Group 1: Stepwise mini-incision mTESE; Group 2: Standard mTESE. The patients’ characteristics of two groups were comparable at baseline

Group 1: Stepwise mini-incision mTESE; Group 2: Standard mTESE

*FSH* Follicle-stimulating hormone, *LH* Luteinizing hormone, T Testosterone

^a^Student’s t test, ^b^Mann–Whitney rank-sum test, ^c^Chi-square test

### Surgical outcomes

The overall SRR was 68.5% (50/73). By comparison of two groups, there were no difference of SRR in total or each side (Table 2). It should be noted that more than half of patients obtained sperm in the initial side. Moreover, the SRR during the initial mini-incision procedure in Group 1 (45.9%, 17/37) was similar with an overall SRR in Group 2 (58.3%, 21/36) (*P* = 0.3). However, in our study, out of the 8 cases who underwent mini-incision TESE and failed to retrieve sperm, none were able to retrieve sperm with standard mTESE. In the standard mTESE group (Group 2), 18 out of 36 patients (50%) underwent bilateral surgery, with 3 successful surgeries and 15 unsuccessful surgeries.

**Table 2:** The sperm retrieval rate in each step of two groups

	**Group 1**			**Group 2**	***P*—value**
		**SRR in each step**	**SRR in total**	**SRR in total**	
**Unilateral procedure**	Step 1	45.9% (17/37)	59.5% (22/37)	50% (18/36)	0.1^a^
	Step 2	13.5% (5/37)			
**Bilateral procedure**	Step 3	8.1% (3/37)	18.9% (7/37)	8.3% (3/36)	0.3^a^
	Step 4	10.8% (4/37)			
**Total**	/	78.4% (29/37)	78.4% (29/37)	58.3% (21/36)	0.1^a^

We initially performed surgery on one testis in both groups, and if no sperm was found, we proceeded with surgery on the contralateral testis. In the mini-incision group, we followed four steps, which were: Step 1: initial mini-incision procedure in the first side; Step 2: standard micro-TESE procedure of the ipsilateral testis; Step 3: mini-incision procedure in the contralateral side; Step 4: standard micro-TESE in the contralateral side

Group 1: Stepwise mini-incision mTESE; Group 2: Standard mTESE

^a^The comparison of SRR in unilateral procedure, bilateral procedure, and total between two groups. Chi-square test

The operation time of the stepwise mini-incision mTESE (72.6 ± 33.9 min) was significantly shorter than that of the standard mTESE (90.4 ± 36.4 min) (*P* = 0). The Johnsen scores from histopathology between two groups did not have significant difference (Group 1: 2 (4, 8) *vs.* Group 2: 2.8 (2, 7), *P* = 0.2).

In addition, two patients need to be noted in Group 2. Both of them were successful in sperm revival, but detected the tissues of fish-like changes in the fixed testes. They were suspected as the testicular tumor, and finally one was diagnosed with right testicular seminoma, while the other was confirmed as a splenogonadal fusion by histopathology. They both underwent testis-preserving surgery with focal lesion excision. There were no malignant lesions or adverse complications during the follow-up.

### The influencing factors of sperm retrieval

We tried to explore the preoperative influencing factors of sperm retrieval and found that only the age at orchidopexy affected the outcomes (Table 3). Patients who underwent orchidopexy before 10 years old possessed a higher SRR than those at older age (*P* = 0). In addition, in successful sperm retrieval cases who were carried an orchidopexy before 10 years old, 80.7% (25/31) were obtained sperm in the initial testes, while only 19.4% (6/31) succeeded in the contralateral surgeries.

**Table 3:** Possible influencing factors of sperm retrieval rate

Parameter	Success	Fail	*P*—value
**Age at orchidopexy [*M*** **(P**_**25**_, P_75_**), years]**	8 (5, 12.75)	14 (7, 28)	0^a^
**Orchidopexy age ≤ 10**	79.5% (31/39)	20.5% (8/39)	0^b^
**Orchidopexy age > 10**	55.9% (19/34)	44.1% (15/34)	
**Mean testicular volume (Mean ± SD, ml)**	7.1 ± 2.9	7.4 ± 2.4	0.7^c^
**FSH (Mean ± SD, IU/L)**	26.9 ± 16.1	34.2 ± 20.1	0.2^c^
**LH (Mean ± SD, IU/L)**	15.0 ± 8.0	17.8 ± 11.5	0.3^c^
**T (Mean ± SD, nmol/L)**	16.2 ± 8.6	13.5 ± 6.2	0.2^c^
**Unilateral cryptorchidism**	24% (12/50)	34.8% (8/23)	0.4^b^
**Bilateral cryptorchidism**	76% (38/50)	65.2% (15/23)	

The parameters previously reported in the literature that may affect the sperm retrieval rate (SRR) are listed. Patients who underwent cryptorchidism surgery before the age of ten had a higher SRR compared to those who underwent surgery after the age of ten, while no statistically significant differences were found for other factors

*FSH* follicle-stimulating hormone, *LH* luteinizing hormone, *T* testosterone

^a^Mann–Whitney rank-sum test, ^b^Chi-square test, ^c^Student’s t test

**Fig. 1:**
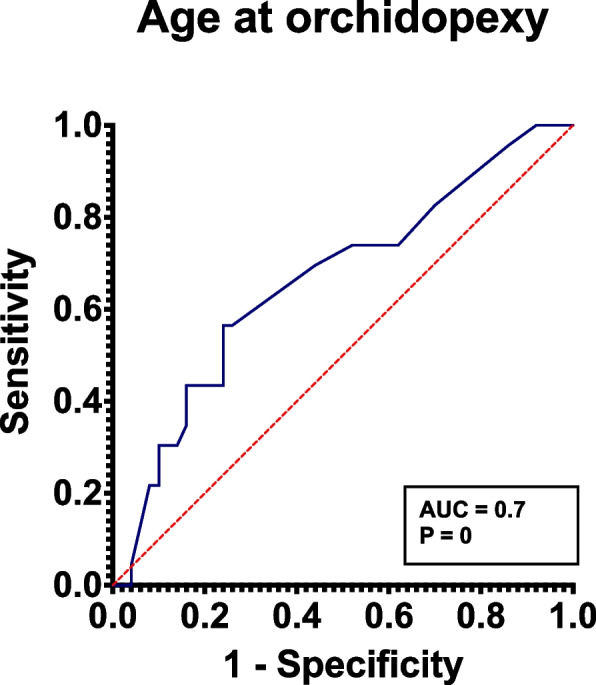
ROC curve of the nomogram for age at orchidopexy. Receiver operating characteristic (ROC) curve for the relationship between age at cryptorchidism surgery and sperm retrieval success rate. The area under the curve (AUC) is 0.7, indicating fair diagnostic accuracy. The diagonal line represents the null hypothesis. The optimal cutoff point, determined by the Youden Index, is indicated by the circle. Sensitivity and specificity are shown for the cutoff point. The P values were calculated using DeLong's test

We further used binary logistics regression analysis of sperm retrieval outcomes about orchidopexy age (χ^2^ = 4.9, *P* = 0, OR (95% CI): 0.945 (0.899–0.994)). Discrimination measurement through ROC curve is shown in Fig. 1, and the AUC for the model was 0.7 (95% CI: 0.534–0.770).

### Postoperative complications

There were no postoperative complications including wound infection, scrotal hematoma, persistent pain, and testicular atrophy during a follow-up period of at least 6 months.

## Discussion

mTESE is an efficient treatment to retrieve sperm from men with NOA, which refers to a careful dissection of the testicular tissue to minimize diminished testicular function [18]. Cryptorchidism, one of the most common pediatric disorders, contributes to a high risk of subfertility [13]. Despite successful orchidopexy in childhood, nearly half of bilateral cryptorchidism and 13% of unilateral cryptorchidism patients later demonstrated NOA [19, 20]. It is precisely because cryptorchidism has caused severe damages to the testes, and the dissections during orchidopexy also have produced potential injuries which may make the testicular state even worse. Therfore, any refinements of mTESE technique may help reduce testicular injuries. In this case–control study, we demonstrated that the stepwise mini-incision mTESE technique is more suitable for NOA patients with a history of cryptorchidism than standard mTESE due to a shorter operation time and minor invasiveness with comparable SRR. In addition, the surgical outcome is superior with a relatively lower orchidopexy age before 10 years old.

It is previously reported that NOA caused by cryptorchidism has a relatively higher SRR than other causes of NOA [15]. In our study, the overall SRR of mTESE was 65.3%, and it is approximately similar to the previous report in the NOA patients with a history of cryptorchidism [21]. There were different hypotheses explaining the high SRR in men with cryptorchidism. The history of bilateral orchidopexy in presumed NOA patients may be a positive predictor for successfully sperm retrieval because of a high prevalence of obstructions and a low probability of other genetic factors [22]. Raman et al.formulated a hypothesis that orchidopexy may have a benefit to preserve the foci of germ cells capable of normal spermatogenesis and could be detected by mTESE [23]. Another explanation is that some of cryptorchidism patients were misdiagnosed in youth when in fact they had retractile testes [24]. In addition, there is no significant difference in SRR between stepwise mini-incision group and standard mTESE group, as such, the introduction of the stepwise mini-incision mTESE did not appear to adversely impact the SRR in our cohort.

To minimize the testicular damage, we aimed to reduce the operation time and effort involved in surgery, and to potentially reduce tissue loss. Zini’s team have reported a mini-incision mTESE approach used in cryptozoospermia and NOA patients [8, 9]. Our team proposed a stepwise mini-incision mTESE procedure and primary proved its clinical value for cryptorchidism NOA patients with a high SRR, and a markedly reduced operation time were also noted [10]. In this study, although a high SRR were reported, most patients did not suffer extensive microdissection and tissue loss. It should be noted that nearly half of the patients (46.0%, 17/37) obtained sperm during the initial mini-incision procedure in stepwise mini-incision mTESE group. Moreover, of those with successful sperm retrieval, a mini-incision and superficial dissection was sufficient to harvest spermatozoa in 58.6% (17/29) cases. The success during mini-incision procedure greatly reduced the difficulties and efforts of mTESE. Interestingly, the SRR of the first step in stepwise mini-incision mTESE group is equal to the overall SRR in standard mTESE group, suggesting that quite a number of patients with successful sperm retrieval should have avoided a wide-incision surgery. However, it should be noted that the sample size factor needed to consider whether there is bias. The operation time was shorter with the stepwise mini-incision mTESE than the standard mTESE. It is partly because rapidly identifying sperm during the mini-incision procedure in stepwise mTESE. In addition, microsurgical closure a 1 cm tunica albuginea incision takes shorter times than a 3–4 cm one [8]. Based on this available data, we inferred that the stepwise mini-incision mTESE technique is a valid approach and perhaps should be the preferred way to potentially minimize testicular injuries.

The optimal age of orchiopexy has been recently recommended between the age of 6 to 12 months [13]. The main goal of this timing is to prevent the impairment of testes, preserve the fertility potential, and decrease the risk of testicular tumors [12]. It is reported that a loss of germ cells begins at around six months of age in boys with cryptorchidism [19, 25]. The age at orchidopexy is closely related to spermatogenesis and endocrine function in adult men [26]. A systematic review drew a conclusion that patients who underwent orchiopexy before the age of ten had a significantly higher SRR than that at an older age [21]. In our study, the SRR was significantly higher in patients who underwent orchidopexy before 10 years old than in patients treated at an older age. In addition, as high as 80.65% of successful patients obtained sperm in the initial testis. The explanation for this result is that testicular growth impairment occurs during puberty in congenitally cryptorchid boys [27]. From another perspective, the orchidopexy should be performed early because postpubertal orchidopexy was associated with approximately double risk of testicular malignancy compared to prepubertal [28]. Unlike children, adults with cryptorchidism are usually recommended to undergo orchiectomy because of increased risk of malignancy, especially in unilateral cases [11]. We performed orchidopexy for adult cryptorchidism patients, part of them had the induction of spermatogenesis after surgery, but not a malignancy had been found. Our results supported the view that postpubertal orchiopexy was capable of restoring fertility by rescuing spermatogenesis.

There were several studies exploring the influencing factors of successful sperm retrieval, including a unilateral or bilateral cryptorchidism history, age at orchidopexy, testicular volume, and serum sexual hormone levels, while many views were controversial [29–32]. The logistics regression and ROC curve analyzes showed that orchidopexy age was a significant predictor to retrieve sperm, even though the predictive utility was not perfect enough probably due to an inadequate sample size. However, there remains not a mature model which can reliably predict the outcome of mTESE. The value of SRR predictors still needs to be further confirmed and optimized by multi-center, large-sample clinical trials.

Two special cases should be emphasized, both of whom underwent orchidopexy at 7 years old and succeeded in sperm retrieval this time. One patient had a splenogonadal fusion, whose abnormal tissue was removed with testicular preservation. Splenogonadal fusion is a rare, frequently misdiagnosed, congenital condition characterized by the fusion of splenic tissue and a gonad or mesonephric remnants. Most splenogonadal fusion is benign, but the risk of malignancy increases when cryptorchidism is present [33]. Many patients undergo unnecessary orchiectomy to evaluate for a testicular tumor. Other patient had a testicular seminoma. mTESE may be helpful for discovering the testicular mass intraoperatively. The testis-preserving tumor resection was performed. This case supported the view that concerns about malignant potential needs to be considered even an orchidopexy has been performed before puberty. The testis-preserving surgical approaches have been increasingly employed in treating benign testicular lesions and may also be considered for management in select cases of malignant testis tumors in a solitary testis [34]. However, we tried to preserve the testis for men who got two testes as these cryptorchid azoospermic men would face greater risks of testicular failure and hypogonadism. During the follow-up, neither of them suffered from testicular mass or tumor recurrence.

This study has several limitations that need to be considered. First, the study design is retrospective and conducted at a single center, which may limit the generalizability of the results to other populations and settings. Second, the sample size is relatively small, which may limit the statistical power of the analysis and increase the risk of selection bias. Third, the study did not evaluate postoperative changes in hormone levels or ultrasonographic findings, which may affect the safety of the mini-incision approach. And then, the study did not record the number of incisions made or the relationship between the number of incisions and successful sperm retrieval, which may impact the interpretation of the results. Moreover, mini-incisions were not allowed for thoroughly exploration so small testicular tumors may be missed. In addition, better designed prospective studies including much more cases are required to validate the clinical value of the stepwise mini-incision mTESE.

## Conclusions

In summary, our study suggests that the stepwise mini-incision mTESE may be a promising approach for sperm retrieval in NOA men with a history of cryptorchidism. While the data indicates that the technique can potentially reduce operation time and minimize surgical invasiveness, further research is needed to validate these findings on a larger scale. The results also highlight the potential impact of age at orchidopexy on SRR, which could have important implications for the management of cryptorchidism.

## Data Availability

The datasets used and/or analyzed during the current study are available from the corresponding author on reasonable request.

## Abbreviations

FSH: Follicle-stimulating hormone
Group 1: Stepwise mini-incision mTESE
Group 2: Standard mTESE
ICSI: Intracytoplasmic sperm injection
LH: Luteinizing hormone
mTESE: Microdissection testicular sperm extraction
NOA: Non-obstructive azoospermia
ROC: Receiver operating characteristic
SRR: Sperm retrieval rate
Step 1: Initial mini-incision procedure in the first side
Step 2: Standard micro-TESE procedure of the ipsilateral testis
Step 3: Mini-incision procedure in the contralateral side
Step 4: Standard micro-TESE in the contralateral side
T: Testosterone
TESE: Testicular sperm extraction
WHO: World Health Organization
